# *Erysipelothrix rhusiopathiae* serotype 5‐associated metritis in a Norwegian Red heifer

**DOI:** 10.1111/apm.12788

**Published:** 2017-11-20

**Authors:** Øyvor Kolbjørnsen, Bjarne Bergsjø, Jeanette Sveen, Tanja Opriessnig

**Affiliations:** ^1^ Department of Pathology Norwegian Veterinary Institute Oslo Norway; ^2^ Romerike Department Norwegian Food Safety Authority Trondheimsvegen Kløfta Norway; ^3^ The Roslin Institute and The Royal (Dick) School of Veterinary Studies University of Edinburgh Midlothian UK; ^4^ Department of Veterinary Diagnostic and Production Animal Medicine College of Veterinary Medicine Iowa State University Ames IA USA

**Keywords:** *Erysipelothrix rhusiopathiae*, metritis, cattle, immunohistochemistry, serotyping

## Abstract

This report summarized the findings of a case of *Erysipelothrix rhusiopathiae* infection in a farmed Norwegian Red heifer located in the south‐east of Norway. The 2.5‐year‐old pregnant heifer was found dead after a short episode of inappetence. On gross exam, the heifer was severely dehydrated with uterine torsion. Microscopically, necrosis of the endometrium was present throughout the uterus along with presence of intralesional Gram‐positive bacteria, interstitial nephritis, and pyelonephritis. *E. rhusiopathiae* was isolated from the uterus and placenta and was also demonstrated by immunohistochemistry (IHC) in the uterus, placenta, and kidney. The *E. rhusiopathiae* isolate was further characterized as serotype 5. To the authors’ knowledge, this is the first report of bacterial metritis associated with *E. rhusiopathiae* serotype 5 infection. The etiology of the infection is unknown but the *E. rhusiopathiae* could have been a primary or opportunistic pathogen. Serotype 5 of *E. rhusiopathiae* has been identified in several mammalian species in recent years and could be emerging.

*Erysipelothrix* spp. is a group of bacteria that can be found worldwide in many species and is of greatest importance for pigs 1. The bacterium is zoonotic and known as an occupational disease in butchers, farmers, and veterinarians among others 1. There are four main *Erysipelothrix* genotypes including *E. rhusiopathiae*,* E. tonsillarum*,* E. species* strain 1, and *E. species* strain 2 2. In cattle, *E. rhusiopathiae*‐associated disease is rare, although subclinical infection is common. Previously antibodies against *E. rhusiopathiae* have been identified in healthy Japanese cattle 3. *E. tonsillarum* was also successfully isolated from tonsils of healthy Japanese cattle at slaughter 4. When 79 *Erysipelothrix* spp. isolates were further characterized, 42 out of 43 typeable isolates were identified as *E. rhusiopathiae* including serotypes 1b, 2, 5, 9, 12, 13, 19, and 21. The remaining isolate belonged to *E. tonsillarum* and was serotype 3 4. *E. rhusiopathiae* has been cultured from vaginal swabs of healthy cattle located in Venezuela 5 and from fecal slurry in cattle herds located in Denmark 6. All these findings indicate that *E. rhusiopathiae* can infect cattle, but this is normally not associated with clinical signs. In this report, we describe a case of metritis associated with *E. rhusiopathiae* infection in a pregnant Norwegian Red heifer with uterine torsion which was confirmed by IHC staining.

## Materials and Methods

### Case history

An 8‐month‐old pregnant, 2.5‐year‐old farmed Norwegian Red heifer with a short episode of inappetence followed by inability to stand and sudden death was submitted for necropsy in February to the Norwegian Veterinary Institute, Oslo, Norway. The heifer was part of a mixed dairy and beef cattle herd located in the south‐east of Norway which consisted of 47 animals. At the time of submission, the beef cattle heifer was strictly housed indoors in a pen with four other heifers. Two other pen‐mates had died 4 weeks earlier, but the cause of death was not determined at that time. No clinical signs were seen in any of the other four heifers. The heifers were fed silage, grain feed, and spent grains in irregular intervals. Water was offered to the animals twice a day.

### Necropsy, histopathology, and immunohistochemistry

Upon arrival at the Norwegian Veterinary Institute, a complete routine gross examination was performed. Samples of all major organs were collected and fixed in 10% formalin for 24 h. Formalin‐fixed tissue sections were processed routinely, embedded in paraffin wax, sectioned, and stained with hematoxylin and eosin (HE). Selected tissue sections were subsequently subjected to Gram staining using routine methods and immunohistochemistry for *E. rhusiopathiae* 7.

### Bacteriology

Samples from different organs were cultured on 5% bovine blood agar plates (Oxoid CM0271 Base No. 2; Oxoid, Basingstoke, UK) and incubated at 5% CO_2_ in an anaerobic atmosphere. In addition, one lactose‐sucrose bromothymol blue agar plate (in‐house), incubated at normal atmosphere, was also used. All plates were incubated at 37 °C for 24 h. Any obtained bacteria were isolated and characterized by Gram staining, light microscopy, stab inoculation of triple sugar iron (TSI) agar and MALDI TOF (MALDI Biotyper, Bruker^®^ Billerica, Massachusetts, USA).

### Characterization of the *Erysipelothrix* isolate

Routine serotyping was performed as described 8. In addition, previously described PCRs to determine the surface protective antigen (Spa) type 9 and genotype 10 were also used.

## Results

### Macroscopic lesions

**Figure 1:**
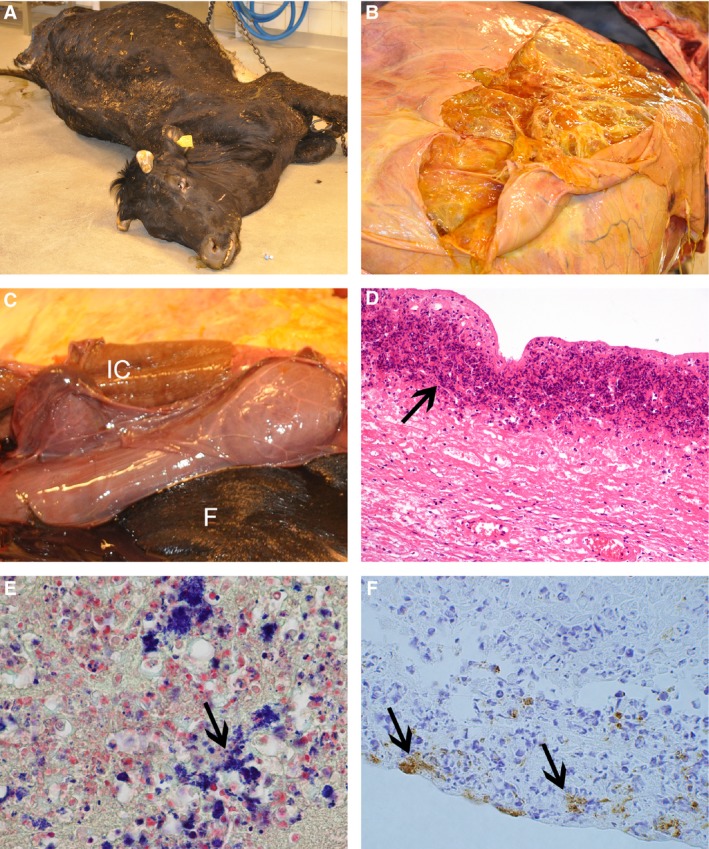
Macroscopic and microscopic lesions in the Norwegian Red heifer infected with *Erysipelothrix rhusiopathiae*. (A) A 2.5‐year‐old pregnant heifer in poor condition with a history of being unable to get up, distended abdomen followed by sudden death was submitted for examination. (B) There are multiple fluid‐filled caverns of various size present on the cut surface of the distended, pregnant uterus. (C) Intercaruncular brown pergament paper‐like tissue (IC) and the fetus (F). (D) Microscopic necrosis of the uterine wall (arrow), HE. (E) Gram staining of the uterine wall indicating the presence of Gram‐positive bacteria, mainly intracellular (arrow). (F) *E. rhusiopathiae*‐like organisms (arrows) present on the placenta surface as demonstrated by immunohistochemistry.

The total weight of the heifer was 465 kg. She was in poor body condition (Fig. 1A), severely dehydrated, had an enlarged abdomen and a bilateral edema in the groin area. The uterus was enlarged, contained a dead fetus, and was twisted by 360 degrees anti‐clockwise. Approximately 50 L of a serous yellow fluid was present in multiple caverns throughout the uterine wall (Fig. 1B). Some caverns also contained blood‐tinged serous fluid or fluid admixed with low amounts of fibrin. The thickness of the uterine wall varied from 1–2 cm up to approximately 10 cm. The intercaruncular endometrium had a pergament paper‐like appearance and was of brown color (Fig. 1C). Many caruncles were enlarged and edematous and some had yellow foci. Multifocally there were fibrinous adhesions between the serosa of the uterus, rumen, and the intestines. The amniotic sac contained several liters of serous, brown amniotic fluid and the placental cotyledons were of brown color. The heifer had pulmonary edema and congestion, and feed‐like material was present in the trachea, bronchi, and lung tissue. The rumen and the abomasum were filled with watery content intermixed with a sparse amount of feed. The small intestine had a watery content, while the large intestine contained normal feces. The gallbladder was dilated and filled with bile, and the texture of the liver was reduced. There were no lesions in the udder; however, all teats had multifocal dry, pergament paper‐like areas. The heart and kidneys were unremarkable. The fetus, a 37 kg bull calf, lying in breech, was of normal appearance, with no specific pathological lesions or malformations.

### Microscopic lesions

Histopathological examination of the uterus revealed multiple areas of fibrin admixed with mainly neutrophils on the serous surface. Cavernous chambers and pockets of different sizes were found throughout the uterine wall, mainly localized within and between the two muscle layers of the myometrium. Some caverns were filled with fluid, others with fluid admixed with various amounts of hemorrhages, fibrin, neutrophils, and macrophages. Few neutrophils, macrophages, and lymphocytes were present throughout the tissue sections, nearby and along the caverns and pockets. In some areas, thrombotic vessels and necrotic debris were present. Multifocal‐to‐confluent necrosis admixed with mainly neutrophils and few macrophages were found in the intercaruncular endometrial mucosa (Fig. 1D). Necrosis, inflammatory cells and hemosiderin‐laden macrophages were also present in some of the caruncles, most prominent at the basal parts, often coalescing with the intercaruncular necrosis. Hyperemia, thrombosis of some vessels, small hemorrhages, neutrophils, and foci with calcification were also seen in the caruncles. The placenta was edematous and a number of unattached cells in the space between the cotyledons and caruncles contained a granular brown hemosiderin negative pigment. Intralesional bacteria were present within the necrotic tissue of the endometrium, the caverns, in the inter‐cavernous tissue, and in the caruncles. Gram‐positive bacteria (Fig. 1E) were often localized in the cytoplasm of macrophages but were also seen in the space between the caruncles and cotyledons, as well as within the cotyledons.

In the kidneys, predominately in cortical areas, there were multifocal interstitial cellular infiltrations of mononuclear cells intermixed with a few neutrophils and mild, multifocal interstitial fibrosis. Occasionally, tubuli were dilated and contained a suppurative exudate (neutrophils) or protein cylinders. The epithelium of all teats had multifocal necrosis with intralesional Gram‐positive coccoid bacteria, and in the stromal tissue, there was suppurative inflammation. The lungs were congested and edematous with multifocal vessels containing small thrombi. In the myocardium, there was mild neutrophilic leukocytosis. Autolytic changes were present in the liver and intestines. There was mild hepatic congestion with mild neutrophilic leukocytosis and scattered Kupffer cells containing bile pigment. The intestinal serosa surface had multifocal fibrin exudation admixed with neutrophils, with a few neutrophils and macrophages scattered in the mesenterium.

### Bacteria identified by culture and immunohistochemistry

*Erysipelothrix rhusiopathiae* was isolated in large numbers from the uterus and the placenta (Table 1). The bacterial flora of the teat was dominated by *Trueperella pyogenes* while there was a mixed non‐specific bacterial flora in the small intestine. *E. rhusiopathiae* antigen was demonstrated in several tissues including uterus, placenta, and kidney by IHC staining (Fig. 1F; Table 1).

**Table 1:** Summary of Gram staining, bacterial isolation and immunohistochemistry (IHC). Organs in which *Erysipelothrix rhusiopathiae* was demonstrated by at least one method are shaded in gray

Organs	Bacterial isolation	Gram stain		IHC^a^
		Result	Comment	
Lung		Positive	Postmortem growth	Negative
Liver		Positive	Postmortem growth	Negative
Kidney		Negative		Positive (low numbers)
Spleen	No significant growth	Negative		Negative
Uterus	*Erysipelothrix* spp.	Positive		Positive (moderate numbers)
Placenta cotyledons	*Erysipelothrix* spp.	Positive		Positive (large numbers)
Placenta between cotyledons		Positive		Positive (low numbers)
Udder		Negative		Negative
Teats	*Staphylococcus* sp.	Positive		Negative
	*Trueperella pyogenes*			
Lymph node udder		Positive	Postmortem growth	Negative
Cervix		Negative		Negative
Rumen		Positive		Negative
Small Intestines	No significant growth	Positive	Postmortem growth	Negative
Dorsal part of cerebrum		Positive		Negative
Heart		Negative		Negative

a On formalin‐fixed tissues using a polyclonal rabbit serum against *E. rhusiopathiae*.

### Further characterization of the *Erysipelothrix rhusiopathiae* isolate

The obtained *E. rhusiopathiae* isolate was identified as *E*. *rhusiopathiae* serotype 5 and contained the SpaA type. The reference strain of serotype 5 is P‐190 and was recovered from a fish 9.

## Discussion

In pigs, it is recognized that *E. rhusiopathiae* can infect pregnant gilts and sows and it has been demonstrated that *E. rhusiopathiae* vaccination reduces the incidence of peripurient vulval discharge, decreases the farrowing intervals and increases the number of life born pigs in breeding herds with clinical disease 11. The bacterium has also been associated with reproductive problems in sows, and bacteria have been isolated from vaginal swabs obtained from unvaccinated sows shortly before parturition 12. In the present case, *E. rhusiopathiae* serotype 5 was identified in the uterus and placenta of a Norwegian Red heifer with uterine torsion and metritis. Previously, *E. rhusiopathiae* has been identified in calves showing hepatic and pulmonary abscesses 13, septicemia and polyserositis 14, or arthritis and polyarthritis 15, 16. In addition, *E. rhusiopathiae*‐associated endocarditis has been reported in a 12‐month‐old Charolaise cross heifer 17.

In the described case involving a Norwegian Red heifer, the precise pathogenicity and sequence of events is unknown. The metritis associated with *E. rhusiopathiae* infection could have been the primary cause of the uterine torsion. Alternatively and more likely, the heifer could have experienced a spontaneous uterine torsion followed by infection with opportunistic bacteria, in this case *E. rhusiopathiae*. In support of the latter option, in a recent study *E. rhusiopathiae* has been identified in vaginal swabs collected from healthy, lactating Criollo Limonero cows and *E. rhusiopathiae* has been suggested to be part of the normal cattle flora 5.

Sources of *E. rhusiopathiae* infection of livestock are often difficult to identify; however, it has been demonstrated that *E. rhusiopathiae* can survive in soil <35 days 18. In this case, the room where the heifer was housed had been used to house pigs 15 years ago. For repair purposes, old wall panels from a different house used for pigs until 2011 had been used in the cattle house. Bacterial isolation on the wall panel surface was negative. Livestock is believed to become infected with this organism through mucous membranes, abrasions, or skin lesions 18. Entry of *E. rhusiopathiae* through a scrotal skin lesion was proposed to be the reason for a local necrotizing inflammation of the testicular tunics in a 2‐year‐old Charolaise bull 19. A 14‐week‐old Holstein heifer developed *E. rhusiopathiae*‐associated lameness after a needle aspiration was attempted 16. The source of the *E. rhusiopathiae* in the present case report remains unknown; however, *E. rhusiopathiae* has been demonstrated to be an opportunistic pathogen and could have been maintained by the resident bovine population on the farm.

Although an old disease, *E. rhusiopathiae* infection appears to be re‐emerging in the domestic pig population 8, 20, 21 and also in the poultry population 22. In recent years, there have been increasing reports of *E. rhusiopathiae* in the wildlife of the Northern hemisphere. For instance, a large outbreak of *E. rhusiopathiae* was associated with increased widespread musk oxen mortality in the Canadian arctic 23. The isolate associated with that outbreak was identified as *E. rhusiopathiae* serotype 5 24 similar to the cow in this case. Furthermore, *E. rhusiopathiae* was also associated with a condition called shaggy lame fox syndrome in wild free‐ranging Pribilof arctic foxes from the Pribilof Islands, Alaska, USA 25. Further characterization of the isolate obtained from the Pribilof arctic foxes in our laboratory indicated an *E. rhusiopathiae* serotype 2 isolate (T. Opriessnig; unpublished). Climate change has been considered by some to impact infectious disease among cold‐adapted animals in Norway 26 and increased temperatures may also be associated with recently reported *E. rhusiopathiae* cases in Alaska, the Artic, and elsewhere. In this case report, the heifer was housed indoors at an ambient temperature and an abrupt temperature change can be ruled out.

## Conclusion

To the authors’ knowledge, this case is the first report of *E. rhusiopathiae* infection in an adult pregnant heifer with metritis in Norway. The isolation of a serotype 5 isolate from a Norwegian Red heifer is in agreement with other studies which demonstrated identification of *E. rhusiopathiae* serotypes 1b, 2, 5, 9, 12, 19, and 21 as well as *E*. species strain 1 serotype 13 in cattle 4. There is likely a range of *E. rhusiopathiae* isolates that can be associated with pathogenicity in various species and future studies should emphasize to collect and characterize isolates associated with unusual clinical manifestations.

The authors wish to thank the laboratory personnel at the Department of Pathology, Norwegian Veterinary Institute, for invaluable technical assistance and Dr. Mette Valheim for taking the gross pictures. TO was supported by the Biotechnology and Biological Sciences Research Council (BBSRC) Institute Strategic Programme Grant awarded to the Roslin Institute (BB/J004324/1; BBS/E/D/20241864).
